# *Drosophila* intestinal stem and progenitor cells are major sources and regulators of homeostatic niche signals

**DOI:** 10.1073/pnas.1719169115

**Published:** 2018-11-07

**Authors:** David P. Doupé, Owen J. Marshall, Hannah Dayton, Andrea H. Brand, Norbert Perrimon

**Affiliations:** ^a^Department of Genetics, Harvard Medical School, Boston, MA 02115;; ^b^Department of Biosciences, Durham University, DH1 3LE Durham, United Kingdom;; ^c^The Gurdon Institute, University of Cambridge, CB2 1QN Cambridge, United Kingdom;; ^d^Department of Physiology Development and Neuroscience, University of Cambridge, CB2 1QN Cambridge, United Kingdom;; ^e^Menzies Institute for Medical Research, University of Tasmania, Hobart, TAS 7000, Australia;; ^f^Howard Hughes Medical Institute, Harvard Medical School, Boston, MA 02115

**Keywords:** epithelial homeostasis, stem cells, niche, microenvironment, insulin

## Abstract

Epithelial homeostasis requires the precise balance of epithelial stem/progenitor proliferation and differentiation. While many signaling pathways that regulate epithelial stem cells have been identified, it is probable that other regulators remain unidentified. Here, we use gene-expression profiling by targeted DamID to identify the stem/progenitor-specific transcription and signaling factors in the *Drosophila* midgut. Many signaling pathway components, including ligands of most major pathways, exhibit stem/progenitor-specific expression and have regulatory regions bound by both intrinsic and extrinsic transcription factors. In addition to previously identified stem/progenitor-derived ligands, we show that both the insulin-like factor Ilp6 and TNF ligand eiger are specifically expressed in the stem/progenitors and regulate normal tissue homeostasis. We propose that intestinal stem cells not only integrate multiple signals but also contribute to and regulate the homeostatic signaling microenvironmental niche through the expression of autocrine and paracrine factors.

## Significance

Most epithelia are turned over throughout adult life as cells are lost from the surface and replaced by the proliferation of stem cells. Precise regulation of stem cells by signals from the local microenvironment or niche is important to maintain epithelial homeostasis. Here, using intestinal stem cells of the *Drosophila* midgut as a model system, we use transcriptome profiling to identify genes expressed specifically in stem and progenitor cells and not their differentiated daughters. We find that stem and progenitor cells express ligands of major developmental signaling pathways to both contribute to the niche and regulate the production of niche signals from other cell types.

**Fig. 1.**
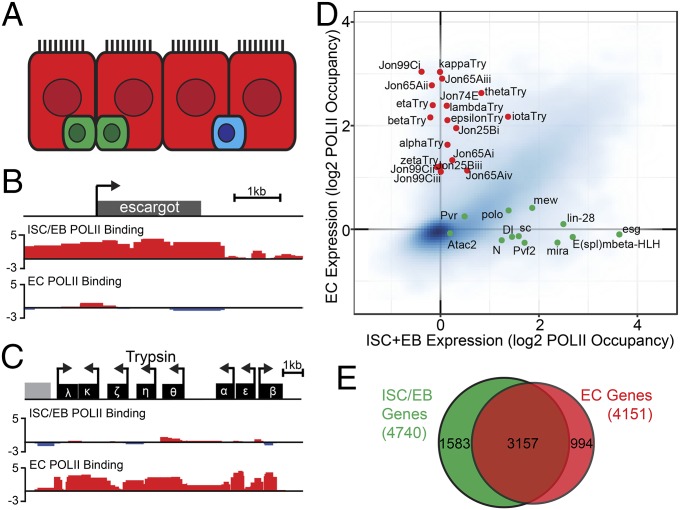
Transcriptome profiling in the midgut by targeted DamID. (*A*) Schematic of midgut epithelial cell types. ISCs and EBs (green), ECs (red), and EEs (blue). ISCs divide to self-renew and generate differentiated EC and EE progeny via differentiating EB progenitors. (*B* and *C*) Example PolII DamID tracks (with unfused Dam control subtracted) for ISC/EB marker *escargot* and EC marker *trypsin* genes. (*D*) Genome-wide gene POLII occupancy in ISC/EBs vs. ECs with known markers highlighted (green for ISC/EB, red for EC). (*E*) Overlap in expressed genes in ISC/EB (green) vs. ECs (red) at FDR < 0.01.

Epithelia are constantly turned over throughout life as cells are lost from the surface and replaced by the proliferation of stem cells. Maintaining epithelial homeostasis is essential, as a failure to replace lost cells may compromise tissue function and an overproduction of cells may lead to cancer. Stem cells’ proliferation and differentiation must therefore be precisely regulated, integrating a range of extrinsic signals to maintain and repair the tissue. Since their identification, *Drosophila* intestinal stem cells (ISCs) have emerged as an excellent model for the study of epithelial stem cells and homeostasis (1, 2). The pseudostratified posterior midgut epithelium consists of just four cell types: proliferating ISCs; differentiating enteroblast progenitors (EBs); absorptive enterocytes (ECs), and secretory enteroendocrine cells (EEs) (Fig. 1*A*). The modes of ISC fate in normal homeostasis and their ability to respond to tissue damage are conserved with mammalian epithelial stem cells (3, 4). Critically, many of the major pathways involved in regulation of mammalian epithelial stem cells—including the EGF, Wnt, Notch, JAK/STAT, Hippo, and insulin pathways—have been shown to regulate *Drosophila* ISCs (5).

While many major pathways are known to be involved in regulation, a comprehensive picture of which signals are involved is lacking. In addition, because many studies use different experimental conditions, such as damaged, growing, or aging midguts, the expression of signaling factors in any given state are not fully characterized. Moreover, because these pathways are used iteratively throughout development, it is clear that context or cell-type–specific factors, such as transcription factors (TFs), are critical to determining cell fate outcomes. Apart from effectors of signaling pathways, relatively few of these—for example escargot (6, 7) and scute (8)—have been identified in the midgut.

Here, we use targeted DamID (9) to profile the transcriptomes of specific cell types in the homeostatic midgut to systematically identify the intrinsic ISC/EB TFs and the expression of signaling molecules. We identify a conserved set of ISC/EB-specific TFs, many of which have orthologs implicated in mammalian epithelial homeostasis or cancer. We then use targeted DamID to identify the targets of one critical ISC/EB TF, Sox21a, and by intersecting its targets with those of a critical extrinsic factor are able to identify key regulators of tissue homeostasis. These include ligands of the major signaling pathways, two of which—eiger and Ilp6—have not previously been implicated in regulation of epithelial homeostasis. We propose that stem and progenitor cells not only integrate a range of signals but are themselves critical sources of signals to maintain their own homeostatic microenvironmental niche.

## Results

### Cell-Type–Specific Transcriptome Profiling of Midgut ISC/EB and EC Cells by Targeted DamID.

Identification of factors that determine stem cell fate requires cell-type–specific profiling of the ISC/EB population. Targeted DamID provides a means to do this in undisturbed, homeostatic tissue without cell isolation that could affect gene expression (9). We profiled both the ISC/EB cells and the predominant differentiated EC cell type, reasoning that factors responsible for stem cell properties would be stem cell-specific, whereas tissue-specific factors and housekeeping genes would be present in both populations. Plotting average methylation across each gene shows a clear separation of genes showing enriched expression in one population or the other (Fig. 1*D*). Known ISC/EB (*escargot*) and EC (*trypsin* gene cluster) genes showed extensive methylation across the gene span only in the escargot and Myo1A populations, respectively (Fig. 1 *B* and *C*). Using a false-discovery rate (FDR) cut-off of 0.01, we identified 4,740 genes expressed in stem/progenitors and 4,151 expressed in ECs. Comparison of these lists showed significant overlap but 1,583 genes were ISC/EB-specific and 994 were EC-specific (Fig. 1*E*; see Dataset S1 for full list).

Gene ontology (GO) analysis shows a clear distinction between terms enriched in the ISC/EB- (terms related to stem cells, proliferation, gene expression, and chromatin) and EC- (membrane transport, metabolism, and proteolysis) specific profiles (*SI Appendix*, Fig. S1 *A* and *B*). This is consistent with their functions as a dynamic and highly regulated stem cell population and an absorptive cell type, respectively. As a further validation, we compiled a list of genes with known, ISC/EB-specific expression in the midgut based on antibody staining, in situ hybridization, or reporters (Dataset S2). Nine of these 16 genes were called as ISC/EB-specific, two were ISC/EB-enriched, four were below significant expression threshold, and only one was detected in both populations. These positive control results compare favorably to published profiles from cell isolation and RNA sequencing (RNA-seq) (10), and overall Spearman’s correlations of 0.58 (ISCs/EBs) and 0.53 (ECs) are observed with RNA-seq data (*SI Appendix*, Fig. S1 *C*–*F*). In addition, high-confidence hits from a genome-wide screen of ISC/EB regulators (11) are enriched in our expression dataset (*SI Appendix*, Fig. S1*G*).

### Identification of ISC/EB-Specific TFs.

ISCs and EBs are regulated by conserved developmental signaling pathways, which are used iteratively throughout development to perform distinct, context-dependent roles. The cell-type–specific transcriptional context is therefore important to determining the outcome of signaling events. While many of the extrinsic signals regulating ISC/EBs have been identified, less is known about the intrinsic, ISC/EB-specific TFs. Identifying these factors would allow a better understanding of the gene regulatory networks in ISC/EBs and profiling their targets may in turn be a means of identifying critical determinants of stem cell properties.

We compared our expression data to published lists of sequence-specific TFs in *Drosophila* from the FlyTF database (12). This identified 101 TFs with ISC/EB-specific expression at the FDR cut-off of 0.01 (Dataset S3). The most highly ISC/EB-enriched TFs (based on fold-difference in average methylation) are shown in Table 1. This unbiased analysis identifies the two best-characterized ISC/EB TFs, escargot (6, 7) and scute (8), along with the circadian rhythm TF cycle, which regulates ISC proliferation (13), and the recently described regulator of ISC differentiation Sox21a (14–16). Other known regulators, such as charlatan (17), and signaling pathway effectors are also identified (Dataset S3). Most of the highly enriched factors have mammalian orthologs [identified using the *Drosophila* RNAi Screening Center Integrative Ortholog Prediction Tool (DIOPT) (18)] that have been implicated in mammalian stem cell fate or carcinogenesis (Table 1). We chose to focus on these, as conservation may imply importance to the conserved process of epithelial homeostasis.

**Table 1.** Stem/progenitor-expressed TFs

Factor	ISC+EB/EC	*Drosophila* midgut (Refs.)	Human ortholog	Mammalian epithelia (Ref.)
Sox21a	6.1	(14–16)	SOX21	(59)
esg	5.2	(1)	SNAI2	(60)
Zfh2	2.8		ZFHX3	(61)
cyc	2.7	(13)	ARNTL	(62)
z	2.6			
apt	2.6		FSBP	
CG11247	2.5		ZNF639	(63)
jumu	2.3		FOXN1	(64)
sc	2.2	(8)	ASCL2	(65)
CG30403	2.0			
Sox100B	2.0		SOX8/9	(66)

TFs (column 1) with FDR < 0.01 in ISC/EBs, >0.01 in ECs and at least twofold difference in POLII occupancy (column 2), and their closest human orthologs (column 4, from DIOPT). References highlight known roles in *Drosophila* (column 3) and mammals (column 5).

**Fig. 2.**
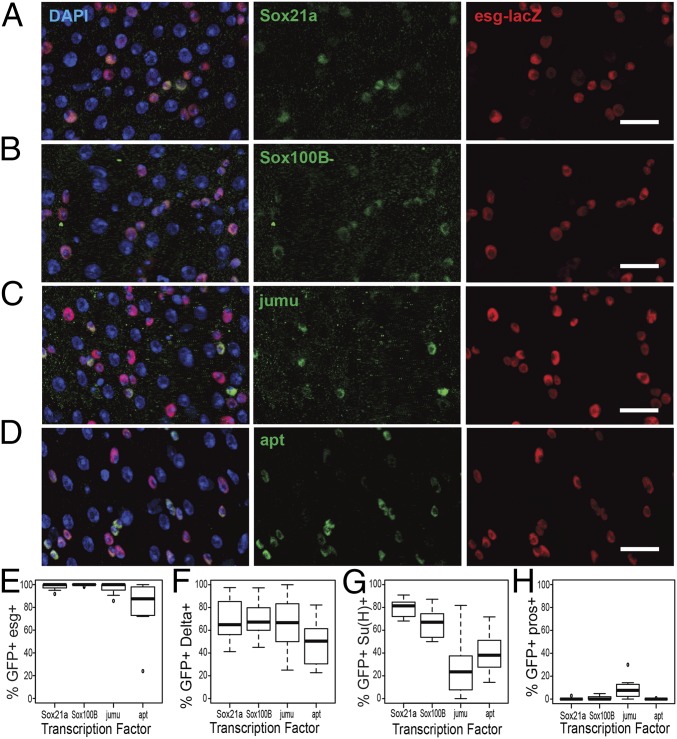
Expression of stem/progenitor-specific TFs. (*A*–*D*) Maximum projection *z*-stacks showing expression of conserved TFs in the midgut. Background signal was subtracted using the remove outliers function (Fiji) and brightness/contrast increased for clarity. DAPI (blue), esg-lacZ (red), and TF (green) for: (*A*) Sox21a-GFP–tagged fosmid, (*B*) Sox100B-GFP trap, (*C*) jumu-GFP protein trap, and (*D*) apt-GFP protein trap. (Scale bars, 20 μm.) (*E*–*G*) Quantification of TF reporter overlap with (*E*) esg-lacZ, (*n* ≥ 10 guts), (*F*) Delta-lacZ (*n* ≥ 9 guts), (*G*) Su(H)-lacZ (*n* ≥ 9 guts), and (*H*) prospero staining (*n* ≥ 7 guts).

Escargot is specifically expressed in ISCs and EBs (1, 2) but the expression patterns of most of the other TFs have not been previously characterized. A Sox21a-GFP fosmid line crossed to an esg-lacZ reporter line showed expression of GFP exclusively in the esg^+^ cells (Fig. 2 *A* and *E*), consistent with recent reports (14–16). GFP protein trap lines for jumu and apontic and a GFP-tagged genomic BAC for Sox100B also showed expression of GFP almost exclusively in the esg^+^ cells (Fig. 2 *B–E*). Further characterization using markers of ISCs, EBs, and EE cells showed that all four factors are expressed in both ISCs and EBs and only jumu showed a small overlap with the EE population (Fig. 2 *F–H* and *SI Appendix*, Fig. S2 *A*–*C*). Both a Zfh2GAL4-enhancer trap driving expression of UAS-EGFP and a ZFh2 antibody specifically labeled small cells of the adult midgut, consistent with ISC/EB-specific expression (*SI Appendix*, Fig. S2 *D* and *E*). Significantly, of these factors only jumu was identified as ISC/EB-specific from previously published RNA-seq data (10).

**Fig. 3.**
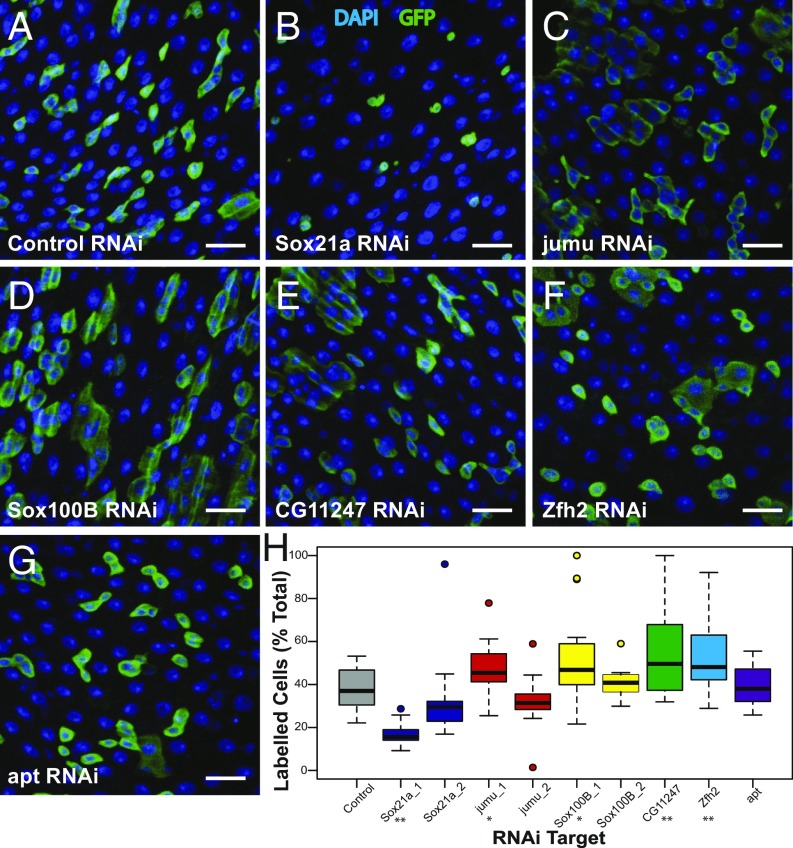
Function of stem/progenitor-specific TFs. (*A*–*G*) Representative projected *z*-stack images of TF RNAi lineage tracing at 10 d postinduction of labeling. DAPI (blue), GFP lineage marker (green). RNAi line as indicated in each panel. (Scale bars, 20 µm.) (*H*) Proportion of labeled cells in posterior midgut at 10 d of TF knockdown. At least 12 guts from at least 3 independent replicates were scored for each condition, *P < 0.05, **P < 0.01 in two-tailed Student’s *t* test.

We used RNAi to knock down each of these TFs with a temperature-inducible lineage-tracing system (19) to identify those that regulate ISC/EB fate. Knockdown of Sox21a resulted in a significant reduction in total cells labeled after 10 d and an absence of large labeled ECs (Fig. 3 *A* and *B*). The number of mitoses per midgut was also significantly reduced on Sox21a knockdown (*SI Appendix*, Fig. S3*A*). This is consistent with recent reports, suggesting that Sox21a is required for both proliferation and differentiation in the posterior midgut (14, 15). Significant increases in the number of labeled cells were observed for jumu, Sox100B, CG11247, and Zfh2 knockdown indicating increased cell production (Fig. 3). There was no significant difference in the proportion of EE cells within the labeled population in any condition, suggesting that while cell production may be increased or decreased the balance of differentiation is maintained (*SI Appendix*, Fig. S3*B*). Consistent with this, in two of the conditions where there is a significant increase in labeled cells (Sox100B and CG11247 RNAi) there is a proportional increase in the percentage of all EE cells that are labeled (*SI Appendix*, Fig. S3*C*).

### Integration of Cell-Type–Specific TF and Extrinsic Pathway Targets Identifies Critical Signaling Molecules.

**Fig. 4.**
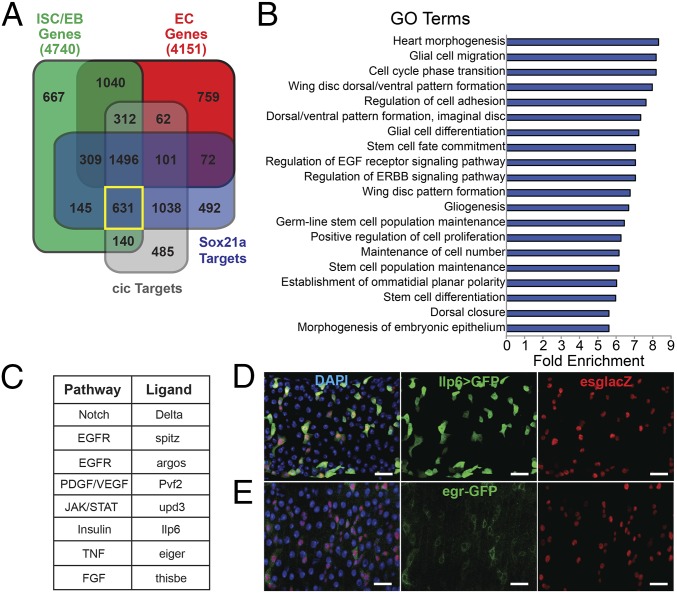
Stem/progenitor-specific TF profiling identifies ligands of major pathways. (*A*) Overlap of genes showing ISC/EB expression (green), EC expression (red), Sox21 binding (blue), and cic binding (gray). See Dataset S4 for gene lists. (*B*) GO terms enriched in the 631 genes specifically expressed in ISC/EBs and with regulatory regions bound by both Sox21a and cic. (*C*) Major developmental signaling pathway ligands specifically expressed in ISC/EBs and bound by both Sox21a and cic. (*D*) Expression of Ilp6 in ISC/EBs. DAPI (blue), EGFP driven by Ilp6-GAL4 (green), and esg-lacZ (red). (Scale bars, 20 µm.) (*E*) Expression of egr in ISC/EBs. DAPI (blue), egr-GFP protein trap (green), and esg-lacZ (red). (Scale bars, 20 µm.)

Sox21a is expressed specifically in ISC/EB and is an important regulator of both differentiation and proliferation in homeostasis. We reasoned that its targets would therefore have important roles in stem cell regulation and used targeted DamID to profile its binding sites in ISCs and EBs. Significant peaks (FDR < 0.01) were identified from the binding profile and were associated with genes where a binding peak overlapped the gene body. The 4,284 target genes identified are listed in Dataset S4. Of these genes, 776 were specifically expressed in ISC/EBs (Fig. 4*A*). A recent study profiled the EGF pathway effector TF capicua (cic) in ISC/EB (20), and because EGF signaling has also been shown to regulate both proliferation and differentiation (20–23), we compared the targets to those of Sox21a. Strikingly, 76% (3,266 of 4,284) of Sox21a targets were also bound by cic; a substantial number of genes may therefore be coregulated by these two TFs and be excellent candidate regulators of stem cell fate (Fig. 4*A* and Dataset S4).

Of the genes, 631 showed ISC/EB-specific expression and were bound by both Sox21a and cic (Fig. 4*A*). GO analysis showed that this set is highly enriched for terms related to stem cells, the cell cycle, or proliferation and signaling (Fig. 4*B*), suggesting that integration of an intrinsic factor and extrinsic input are indeed suitable criteria to identify critical regulators. Almost all of the most highly enriched ISC/EB TFs (Fig. 2*A*) fall into this category and many signaling pathway components were also included.

We noted that at least one ligand of most major signaling pathways showed ISC/EB-specific expression and was bound by both Sox21a and cic (Fig. 4*C*). Whole-gut qPCR showed changes in expression of most of these factors in response to either Sox21a overexpression or knockdown, Ras overexpression, or both (*SI Appendix*, Fig. S4 *I* and *J*), consistent with direct regulation, but the lack of cell-type specificity means indirect effects cannot be ruled out. Some of these ligands had been previously implicated in epithelial homeostasis and have known ISC/EB expression: the Notch ligand Delta regulates differentiation (24); Pvf2 is an autocrine factor that promotes ISC maintenance (25); and spitz regulates proliferation (21). The cytokine upd3 is known to regulate ISC homeostasis in response to damage (19, 26). In normal homeostasis it has been described as having expression predominantly in ECs, but we also observe sporadic expression in some small ISC/EBs (*SI Appendix*, Fig. S4*J*); expression is therefore broader than DamID suggests. In addition, three other major pathway ligands—Ilp6, eiger (egr), and thisbe—also follow the same pattern of ISC/EB expression and integration of regulation. We validated the expression of the first two of these in ISC/EBs using an Ilp6-GAL4 line (27) driving UAS-EGFP (Fig. 4*E*) and an egr-GFP protein trap (28), and found near complete overlap with esg-lacZ. Of the Ilp6^+^ cells, 97.5 ± 4.7% (mean ± SD), and of the egr^+^ cells, 94.5 ± 6.3% were also esg-lacZ^+^ (*SI Appendix*, Fig. S4 *G* and *H*). Reciprocally, 85.9 ± 14.4% of all esg^+^ cells were Ilp6^+^, suggesting expression in both ISCs and EBs, and we observe Ilp6 expression in both of these cell types (*SI Appendix*, Fig. S4 *A* and *B*). The percentage of esg^+^ cells that were also egr^+^ was lower and more variable (48.4 ± 27%), which could indicate expression in either ISCs or EBs, but significant expression was seen in both cell types (*SI Appendix*, Figs. S4 *D*, *E*, and *H*). These results suggest that egr is expressed either in a subset of ISCs and EBs or has variable expression levels, depending on the local environment. Neither Ilp6 nor egr showed significant expression in prospero^+^ EE cells (*SI Appendix*, Fig. S4 *C* and *F*–*H*).

### Ilp6 and egr Are ISC/EB-Derived Regulators of Epithelial Homeostasis.

**Fig. 5.**
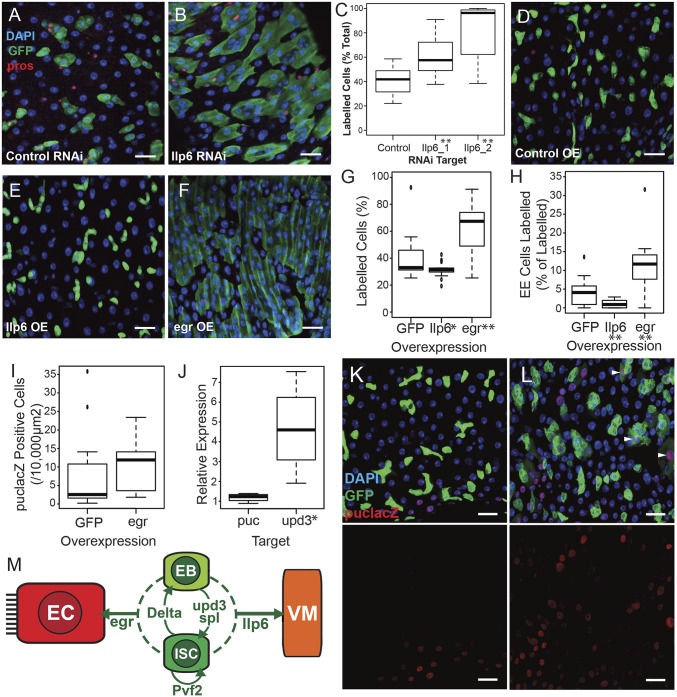
Ilp6 and egr regulate epithelial homeostasis. (*A*–*C*) Knockdown of Ilp6 increases tissue turnover. Lineage tracing (GFP, green) shows a significant increase in cell production on Ilp6 knockdown (*B*) compared with control luciferase RNAi (*A*) (blue is DAPI, red is EE marker prospero) as quantified in *C* (*n* ≥ 17 guts, **P < 0.01 in two tailed Student’s *t* test). (Scale bars, 20 µm.) (*D*–*H*) Overexpression of egr increases epithelial turnover. Overexpression of egr (*F*) increases cell production compared with control RNAi (*D*) and Ilp6 overexpression (*E*), quantification in *G* (*n* ≥ 15 guts, *P < 0.05, **P < 0.01 in two tailed Student’s *t* test). EE cell production is reduced on Ilp6 overexpression (*E*) and increased on egr overexpression (*F*) as quantified in *H* (*n* ≥ 15 guts, **P < 0.01 in two tailed Student’s’ *t* test). (Colors and scale bars as in *A* and *B*.) (*I*, *K*, and *L*) egr overexpression affects the expression of a puc-lacZ reporter (red), particularly inducing expression in GFP^+^ cells (white arrowheads). DAPI is blue, GFP driven by esg-GAL4 is green. (Scale bar: 20 µm.) (*J*) egr overexpression increases whole-gut expression of cytokine ligand upd3 by qPCR. *P < 0.05. (*M*) Schematic of known ISC/EB-derived signals (for references, see main text) showing ISCs (dark green), EBs (light green), ECs (red), and visceral muscle (VM, orange). Arrows indicate known (solid lines) or proposed (dashed lines) target cell types.

The insulin signaling pathway has been shown to positively regulate stem cell proliferation, EB differentiation (29), and feeding-driven expansion of the young gut (30). However, the only insulin ligand known to function in the gut is Ilp3, which is expressed in the surrounding visceral muscle (30). Because insulin pathway activation in ISC/EB drives proliferation and differentiation, we hypothesized that Ilp6 may be an autocrine factor promoting proliferation and differentiation. However, knockdown of Ilp6 by RNAi resulted in increased proliferation and differentiation (Fig. 5 *A–C* and *SI Appendix*, Fig. S5*A*). In addition, overexpression reduced both the total labeled cell number and the proportion of labeled cells that expressed an EE marker in a lineage-tracing experiment (Fig. 5 *D*, *E*, *G*, and *H*). Ilp6 is an insulin receptor agonist but has been shown in other contexts to work in opposition to the major circulating Ilps (31, 32). On starvation, Ilp6 is induced in the fat body and acts on the insulin-producing cells to down-regulate Ilp2 and Ilp5. To test whether a similar mechanism may function in the gut, we performed whole-gut qPCR for Ilp3 when Ilp6 is knocked down specifically in ISC/EBs. Consistent with a negative feedback role, we found that Ilp3 was up-regulated upon Ilp6 knockdown (*SI Appendix*, Fig. S5*E*). Additional studies will be needed to determine the mechanism of this regulation and address the interesting question of how different Ilps can have opposing effects.

In contrast to Ilp6, overexpression of egr resulted in a significant increase in tissue turnover (Fig. 5 *D* and *F*), with both the total number of cells produced (Fig. 5*G*) and number of differentiated cells produced (Fig. 5*H*) significantly increased. Knockdown of egr had no significant effect on the rate of cell production but may impact upon differentiation rate (*SI Appendix*, Fig. S5 *A* and *F*). Because egr is a ligand for the JNK signaling pathway, we examined the expression of the JNK reporter, puc-lacZ, on ISC/EB overexpression of egr (Fig. 5 *I* and *J*). In normal homeostasis, puc-lacZ expression is usually seen in small numbers of large ECs in the posterior midgut. Overexpression of egr increased the average number of puc-lacZ^+^ cells per area of the posterior midgut (Fig. 5 *I–K*). In particular, the majority of wild-type gut images contained no puc-lacZ^+^ cells within the esg > GFP population (7 of 11), whereas almost all images of egr overexpression guts contained double positives (9 of 10) (*P* < 0.05, χ^2^ test). Many of these cells have relatively faint GFP expression, consistent with perdurance into newly differentiated ECs, suggesting that egr may activate JNK signaling in differentiating EBs and newly generated ECs. Whole midgut qPCR upon egr overexpression did not show up-regulation of puc RNA, which may be due to the increased sensitivity of accumulating stable β-gal or regional differences in the gut. Previous studies have only shown a small increase in puc expression by qPCR even in damaged gut (19). We do, however, see an increase in expression of the cytokine ligand Upd3, which has been shown to be a target of JNK signaling in the ECs (Fig. 5*L*).

## Discussion

### Transcriptional Regulation of Epithelial Stem Cells.

The regulation of stem cell fate depends on the integration of autonomous intrinsic factors and extrinsic signals at the transcriptional level. We have used targeted DamID to profile ISC/EB-specific gene expression and identify a set of TFs whose function may be conserved to mammalian stem cell systems. Further exploration of the transcriptional network downstream of these factors may therefore prove useful in understanding intrinsic stem cell regulation across a range of systems. We characterized the targets of one critical regulator, Sox21a, and, by overlapping its targets with those of an extrinsic signal and our expression data, have been able to identify a set of genes highly enriched for regulators of stem cell fate. Our genome-wide datasets constitute a robust resource for future work; for example, Kim et al. (33) used our datasets to focus screens on expressed genes.

### Insulin-Like Factors and TNF Ligands in Stem Cell Niches.

We identify a set of ligands for the major homeostatic signaling pathways that are expressed in the ISC/EBs and have their regulatory regions bound by both Sox21a and cic. Some of these have already been characterized in the midgut but we identified two previously uncharacterized ligands, the insulin-like protein Ilp6 and the TNF ligand egr. Insulin signaling, in the form of visceral muscle Ilp3 downstream of systemic Ilp2 has previously been shown to positively regulate both EB differentiation and ISC proliferation (17, 29, 30, 34). Strikingly, the ISC/EB-derived Ilp6 acts as a negative regulator of tissue turnover, working in opposition to the Ilp3 niche signal. A similar Ilp6 negative feedback loop functions between the fat body and insulin-producing cells on starvation, with Ilp6 repressing the expression of Ilp2 and Ilp5 and, hence, organism-wide insulin signaling (31). Additional work will be required to establish how Ilp6, which functions as a positive insulin receptor ligand (27, 35), is able to function in this way without triggering autocrine insulin pathway activity.

One possible explanation may be differences between Ilp6 and Ilp3 in receptor binding affinity or strength of activation upon binding. Alternatively, reciprocal regulation between the different Ilps and compensatory up-regulation of other Ilps, as has been observed in other systems (36, 37), may result in a higher level of total Ilps when Ilp6 is knocked down. Another possibility is that Ilp binding proteins may influence the local activity or range of the Ilps in the gut. For example, Impl2, which is highly up-regulated in Yki gut tumors (38), is expressed by ISC/EBs (Dataset S1). The affinity of Impl2 for Ilp6 has not been tested, but some evidence suggests that while Impl2 binds Ilp2 and Ilp5, it may not bind Ilp3 (39). Coexpression of Impl2 may therefore differentially affect the function or range of Ilp6 and Ilp3.

TNF signaling had not previously been implicated in homeostasis of the *Drosophila* midgut, but the downstream JNK pathway plays an important role in ECs in response to a range of stress signals. egr expression is up-regulated in response to ISC/EB Ras overexpression and promotes turnover through feed-forward signaling to differentiated ECs. In mammalian intestinal epithelium, tumor necrosis factors play a range of roles (40) and the receptor TNFR2 is involved in hyperplasia and chronic inflammation (41). The closest mammalian ortholog of egr, EDA, is an important regulator of epidermal appendage development (42, 43), suggesting some functional conservation of TNF signaling in mammalian epithelia. Similarly, insulin-like growth factors play important roles in the regulation of mammalian colon stem cells in normal homeostasis, colorectal cancer, and diabetic enteropathy (44–46).

### Stem and Progenitor Cells as Active Contributors to and Regulators of the Niche.

Since the proposal of the niche concept in the hematopoietic system (47), stem cell niches have been characterized in a range of tissues, from *Drosophila* to mammals. Epithelial stem and progenitor cells of the mammalian hair follicles and intestinal crypts, and stem cells of the *Drosophila* germline and midgut, are precisely regulated by signals from the local microenvironment to meet the needs of the tissue (48–51). We have found that in the *Drosophila* midgut the ISCs and their differentiating EB daughter cells not only receive signals from the surrounding cells that constitute their niche, but are themselves major sources of signals for many pathways (Fig. 5*M*). This provides a mechanism whereby the stem cells may actively feed back or forward to fine-tune the signaling balance and respond rapidly to challenges. Indeed, manipulation of ISC/EB ligand levels impacts the expression of ligands from the surrounding cells that form the niche. Many of these ligands are also expressed in other tissues and further work will be needed to understand the relative contributions that ligands from more distant sources may make.

Recent studies on mammalian airway stem cells have suggested that such stem/progenitor-derived signals may play an underappreciated role in tissue homeostasis (52, 53). Autocrine signals from stem cells have been identified as critical stem cell regulators in a range of systems, including Wnt ligands and antagonists in mammalian interfollicular epidermis (54) and Pvf2 in *Drosophila* ISCs (25). Signals from stem cells to regulate the niche have been identified in both *Drosophila* and *Caenorhabditis elegans* germlines (55, 56). The ability of stem cells to contribute substantially to their own niche has significant implications for tumorigenesis as misregulation could be a means to drive overgrowth. Similarly, in the context of metastasis, colonization of new niches can involve instructive signals from tumor cells to remodel or reprogram signaling in their new metastatic niches (57, 58).

Our findings suggest that just as the tissue is maintained in a dynamic state of homeostatic turnover, the local signaling microenvironment is likely highly dynamic, incorporating stem cell responses to extrinsic signals. Further studies that allow the spatiotemporal dynamics of signaling pathway activation and ligand expression will be important to dissect the relationships that maintain a balanced signaling state and to understand how these may be perturbed in cancer development and progression.

## Materials and Methods

Detailed materials and methods, including fly stocks and crosses, cloning and transgenic fly generation, staining and imaging, qRT-PCR, and targeted DamID experiments and analysis are included in *SI Appendix*. Sequencing files are available from the Gene Expression Omnibus (accession no. GSE101814).

## Supplementary Material

Supplementary File

Supplementary File

Supplementary File

Supplementary File

Supplementary File
