# Supplementary material for: Drosophila intestinal stem and progenitor cells are major sources and regulators of homeostatic niche signals
Source: Proc Natl Acad Sci U S A. 2018 Nov 7;115(48):12218–23. doi: 10.1073/pnas.1719169115 (PMC6275525; doi:10.1073/pnas.1719169115)
Supplement: Supplementary File [file pnas.1719169115.sapp.pdf]

PNAS
www.pnas.org

Supplementary Information for

Drosophila intestinal stem and progenitor cells are major sources and regulators of homeostatic niche signals

David P. Doupé[a,b,1], Owen J. Marshall[c,d,e], Hannah Dayton[a], Andrea H. Brand[c,d], and Norbert Perrimon[a,f,1]

[a] Department of Genetics, Harvard Medical School, Boston, MA 02115

[b] Department of Biosciences, Durham University, DH1 3LE Durham, United Kingdom;

[c] The Gurdon Institute, University of Cambridge, CB2 1QN Cambridge, United Kingdom

[d] Department of Physiology Development and Neuroscience, University of Cambridge, CB2 1QN Cambridge, United Kingdom

[e] Menzies Institute for Medical Research, Hobart, TAS 7000, Australia

[f] Howard Hughes Medical Institute, Harvard Medical School, Boston, MA 02115

[1] To whom correspondence should be addressed.
Email: david.p.doupe@durham.ac.uk, perrimon@receptor.med.harvard.edu

This PDF file includes:

Figs. S1 to S5
Legends for Supplementary Datasets S1 to S4
Supplementary Methods

www.pnas.org/cgi/doi/10.1073/pnas.1719169115

Supplementary Information

Fly stocks and crosses

The following fly stocks were used (BL indicates Bloomington *Drosophila* Stock Center, VDRC is Vienna *Drosophila* RNAi Collection): *esg-GAL4, UAS-GFP, tub-GAL80^{ts}* (1); *Myo1A-GAL4, tub-GAL80^{ts}* (2); *UAST-LT3-NDam* and *UAST-LT3-NDam-PolII* (3); *UAST-LT3-NDam-Sox21a* (this study); *UAST-Sox21a* (this study) ;*UAS-Ras* (wild-type) (BL5788); *UAS-EGFP* (on III lab stock); *UAS-Ilp6* (Leopold Lab,(4)); *UAS-egr* (Perrimon lab stock from Sara Cherry); *esg-lacZ* (BL10359); Su(H)-lacZ; Dl-lacZ; *Sox21a-GFP* (VDRC318496,(5)); *jumu-GFP* (BL 51534 (CC00294)); *apt-GFP* (BL 51550 (CC01186); Sox100B-GFP (BL 66743); *Zfh2-GAL4* (BL 25676); *esg-GAL4, UAS-GFP, tub-GAL80^{ts}; actin>CD2> GAL4, UAS-flp* ((esg^{ts}F/O)(2)); *UAS-Luc RNAi* (BL31603); *UAS-Sox21a RNAi* (BL31902, BL53991); *UAS-jumu RNAi* (BL35438, VDRC44117); *UAS-Sox100B RNAi* (BL35656, BL57417); *UAS-CG11247 RNAi* (BL31947, VDRC52116); *UAS-Zfh2 RNAi* (BL50643); *UAS-apt RNAi* (BL26236); *UAS-Ilp6 RNAi* (BL31379, BL33684); *UAS-egr RNAi* (BL58993, BL55276); *Ilp6-GAL4* (6); *egr-GFP* (MI15372-GFSTF, Bellen lab); *puc-lacZ* (7). Flies were maintained on standard cornmeal/ yeast at 25°C with 12 hours light dark cycles unless otherwise stated. Mated adult females, age matched to within 72 hours, were used in all experiments at consistent densities and 2:1 female to male ratios. For temperature inducible experiments flies were maintained at 18C for 3-8 days and then shifted to 29C for the times stated.

Cloning and transgenic fly generation

Full length *Sox21a* was amplified from cDNA with phusion polymerase using attagcggccgctgatgacgagcatctcggcc forward and attactcgagtcaaatgatgtttggcggact reverse primers and cloned into pUAST-LT3-NDam and pUAST-attB with the NotI and XhoI restriction sites (NEB). The Dam construct was injected into *yw;;attP2* (Bestgene) and overexpression construct into *yv;; attP40* (lab stock).

Staining and imaging

Whole midguts were dissected in PBS on ice and fixed in 4% PFA in PBS at RT for 30 minutes. Fixed guts were washed 3x PBS, blocked in 1%BSA, 0.5%Triton, 5%NDS in PBS for 30 min at RT and incubated with primary antibodies overnight at 4°C in PBS 0.5% triton. Guts were then washed 3x10min PBS and incubated with appropriate secondary antibodies for 2 hours at RT, incubated with DAPI (1/2000 of 1mg/ml stock) in PBS 0.5% triton, washed 3x 10min PBS and mounted in vectashield. Primary antibodies used were: anti-GFP (Rb 1/2000 Molecular Probes A6455, Mse 1/500 Molecular Probes A11120), anti-GFP (Ckn, Abcam ab13970 1/1000), anti-βgal (Rb 1/10000, Cappell), anti-Pros (Mse 1.100 DSHB MR1A). Secondary antibodies were anti rabbit, mouse, rat or chicken raised in donkey, conjugated to alexa fluor 488 or 555 from Molecular Probes. Z stack images were acquired from the posterior midgut on a Zeiss LSM 780 or Leica SP5 Confocal microscope with a 40x oil immersion lens using identical acquisition conditions for all samples from a given replicate of a given experiment.

qRT-PCR

15 midguts per samples were dissected in PBS on ice and RNA was extracted using trizol reagent and purified with an RNA easy kit. cDNA was generated with iScript reverse transcriptase kit using 1ug of RNA as template. qRT-PCR was performed using SYBR Green on a Bio-Rad CFX96 system. Analysis was performed using Bio-Rad CFX software and the ΔΔCq method using GAPDH as a control. Primers used for qRT-PCR were:

GAPDH_F CCAATGTCTCCGTTGTGGA
GAPDH_RTCGGTGTAGCCCAGGATT
upd3_F CTGGTCACTGATCTTACTCGCC
upd3_R GGATTGGTGGGATTGATGGGA
puc_F CATGTGGCTAGCAATTTGA
puc_R GTCTTCGAAAAACGTACAGC
Delta_F AATCCCATCCAGTTCCCCTTC
Delta_R ATTGCCGCTGTTGTTCGTATC
spitz_F TGCGGTGAAGATAGCCGATC
spitz_R TTCGCATCGCTGTCCCATAA
argos_F TGCTGTTGGGTGAATTTCAGG
argos_R CGACTGGTCCAGATGATCCA
Pvf2_F GGTGGTCCACATCACGAGAG
Pvf2_R CGACTTTGTCGCTGCATCTG
Ilp6_F CGATGTATTTCCCAACAGTTTCG
Ilp6_R AAATCGGTTACGTTCTGCAAGTC
eiger_F AGCTGATCCCCCTGGTTTTG
eiger_R GCCAGATCGTTAGTGCGAGA
ths_F CGTCCGCAACAACCATGAAG
ths_R CATTGCGCACATAGGTCAGC
Ilp3_F AGAGAACTTTGGACCCCGTGAA
Ilp3_R TGAACCGAACTATCACTCAACAGTCT

Targeted DamID Experiments

esg-GAL4^{ts} and *Myo1A-GAL4^{ts}* flies were crossed to *UAST-LT3-NDam* and *UAST-LT3-NDam-PolII* flies at 18 degrees. Progeny were collected in 48 hour batches and aged for a further 3 days at 18 before transfer to 29 degrees to induce Dam protein expression for 24 hours. 40 midguts per condition were dissected in cold PBS and stored at -80C. Methylated fragments were isolated and next generation sequencing libraries were prepared as described previously(3, 8) and below.

DamID Library Preparation, Sequencing and Data Analysis

Genomic DNA was isolated using a DNeasy Blood and Tissue kit (Qiagen) and digested

overnight with DpnI. Adapters were then ligated using T4 DNA ligase (Roche) for 2 hours at 16ºC and ligated products were digested with DpnII. Ligated fragments were enriched by 17 cycles of PCR amplification using Advantage DNA polymerase (Clontech). DamID adaptors were then removed by digestion with AlwI. For PolII and Control experiments sample shearing (Covaris), Illumina library preparation (PrepX ILM Kit on an Apollo 324) and sequencing (Illumina HiSeq 2500, 50bp single reads, v3 chemistry) were performed at the Bauer Core Facility, FAS Division of Science, Harvard University. For Sox21a and associated control library preparation, samples were sonicated in a Bioruptor Plus (Diagenode) to reduce the average DNA fragment size to 300bp, and DamID adaptors were removed via overnight AlwI digestion. The resulting DNA was purified via magnetic bead clean-up using Seramag beads42 in 20% (w/v) PEG-8000, and 500ng of DNA was processed for Illumina Sequencing. 50bp single-end reads were obtained via a HiSeq 1500 (Illumina) as previously described(8, 9). Libraries were multiplexed such as to yield at least 20 million mapped reads per sample. Sequencing files are available from GEO (GSE101814).

Analysis of DamID data was performed as previously described(8, 9). FASTQ data from Cic DamID data was obtained from GSE74188 (10). Rscripts for the complete data analysis pipeline are available at https://github.com/AHBrand-Lab. NGS reads in FASTQ format were aligned and processed using damidseq_pipeline 1.4 with default parameters. The resulting gatc.gff ratio files were averaged for each dataset. RNA Pol II (RPII215) datasets were processed with the polii.gene.call script (available at https://github.com/AHBrand-Lab) to call genes with significantly enriched RNA Pol II occupancy (FDR < 0.01). For Sox21a and Cic binding data, peaks were called from averaged GFF ratio files using find_peaks with –gene_pad=0, with genes associated with peaks if a binding peak overlapped the gene body. Processed RNAseq data was obtained from GSE61361. All other analyses were performed using R (www.r-project.org).

Data Analysis and Figure Preparation

Gene ontology PANTHER Over-representation analysis was performed using GO Ontology database Release 2016-11-30 at geneontology.org(11, 12). Ortholog identification was performed using DIOPT(13), with the highest scoring orthologs included in Figure 2A. Venn diagrams were initially plotted using Biovenn(14). Figures were assembled in Adobe Illustrator. Microscope images were processed in Fiji(15). Unprocessed Z stacks were max projected for quantification and figures. Background signal was subtracted using the remove outliers function and brightness adjusted (equally across comparable images) where necessary for figure panel clarity. Plots were generated using R studio, with the exception of 1D. Statistical analysis was done in Microsoft Excel unless otherwise stated.

Supplementary References

1. Micchelli C a, Perrimon N (2006) Evidence that stem cells reside in the adult Drosophila midgut epithelium. *Nature* 439(7075):475–9.
2. Jiang H, et al. (2009) Cytokine/Jak/Stat signaling mediates regeneration and homeostasis in the Drosophila midgut. *Cell* 137(7):1343–55.
3. Southall TD, et al. (2013) Cell-Type-Specific Profiling of Gene Expression and Chromatin Binding without Cell Isolation: Assaying RNA Pol II Occupancy in Neural Stem Cells. *Dev Cell* 26(1):101–12.
4. Ikeya T, Galic M, Belawat P (2002) Nutrient-Dependent Expression of Insulin-like Peptides from Neuroendocrine Cells in the CNS Contributes to Growth Regulation in Drosophila. *Curr Biol* 12(15):1293–1300.
5. Sarov M, et al. (2016) A genome-wide resource for the analysis of protein localisation in Drosophila. *Elife* 5:1–38.
6. Chell JM, Brand AH (2010) Nutrition-responsive glia control exit of neural stem cells from quiescence. *Cell* 143(7):1161–73.
7. Martín-Blanco E, et al. (1998) puckered encodes a phosphatase that mediates a feedback loop regulating JNK activity during dorsal closure in Drosophila. *Genes Dev* 12(4):557–670.
8. Marshall OJ, Southall TD, Cheetham SW, Brand AH (2016) Cell-type-specific profiling of protein-DNA interactions without cell isolation using targeted DamID with next-generation sequencing. *Nat Protoc* 11(9):1586–1598.
9. Marshall OJ, Brand AH (2015) Damidseq-pipeline: An automated pipeline for processing DamID sequencing datasets. *Bioinformatics* 31(20):3371–3373.
10. Jin Y, et al. (2015) EGFR/Ras Signaling Controls Drosophila Intestinal Stem Cell Proliferation via Capicua-Regulated Genes. *PLoS Genet* 11(12):1–27.
11. The Gene Ontology Consortium (2015) Gene Ontology Consortium: going forward. *Nucleic Acids Res* 43(D1):D1049–D1056.
12. Ashburner M, et al. (2000) Gene ontology: tool for the unification of biology. The Gene Ontology Consortium. *Nat Genet* 25. doi:10.1038/75556.
13. Hu Y, et al. (2011) An integrative approach to ortholog prediction for disease-focused and other functional studies. *BMC Bioinformatics* 12:357.
14. Hulsen T, de Vlieg J, Alkema W (2008) BioVenn -- a web application for the comparison and visualization of biological lists using area-proportional Venn diagrams. *BMC Genomics* 9(1):488.
15. Schindelin J, et al. (2012) Fiji: an open-source platform for biological-image analysis. *Nat Meth* 9(7):676–682.

Supplementary Dataset Legends

Dataset S1 - Full list of expressed genes at FDR <0.01 in each population

Dataset S2 - Expression of positive control genes (values given only for those with significant expression).

Dataset S3 - Stem/progenitor enriched TFs

Dataset S4 - Sox21a targets and overlap with cic

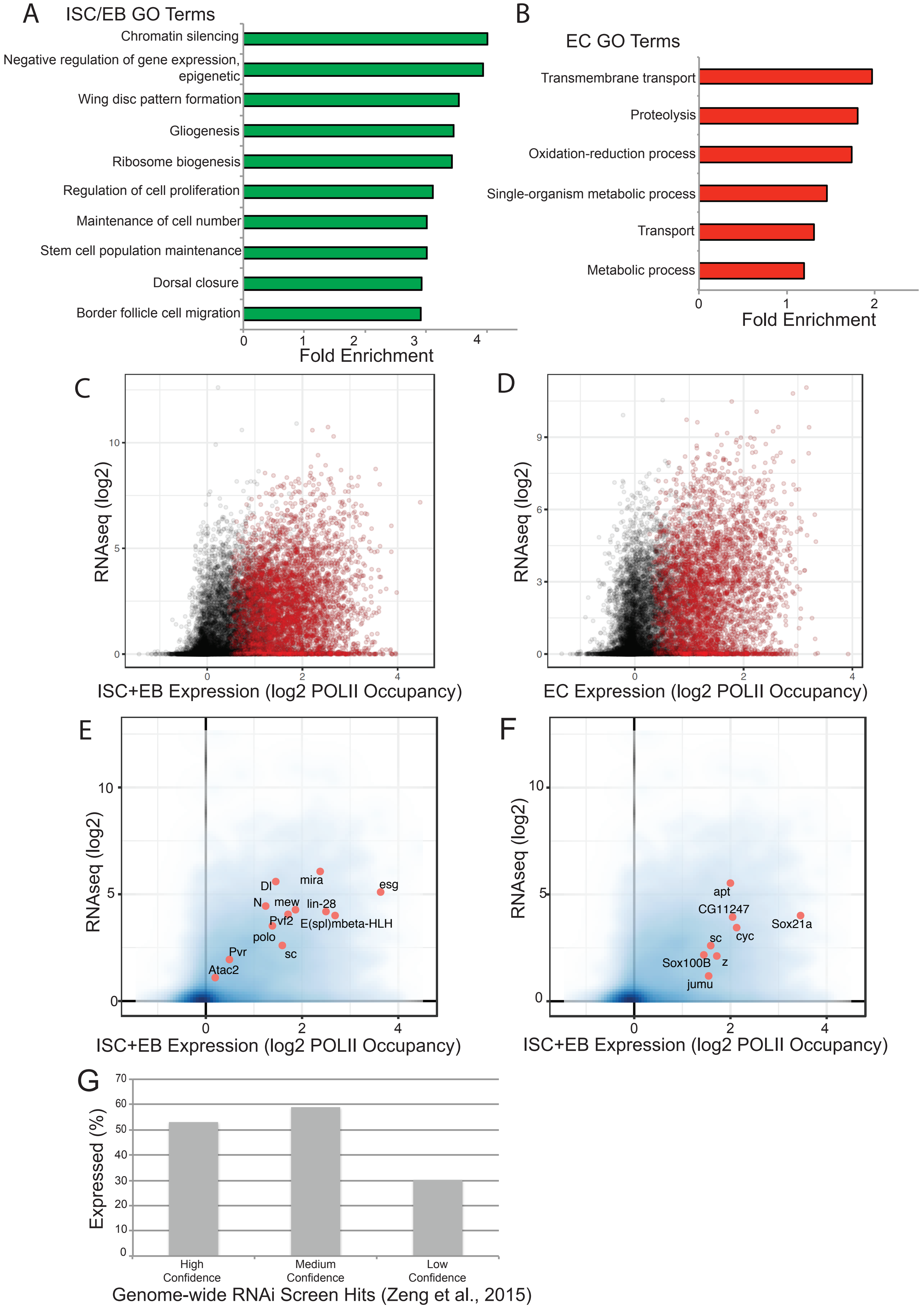
A
ISC/EB GO Terms
Chromatin silencing
Negative regulation of gene expression, epigenetic
Wing disc pattern formation
Gliogenesis
Ribosome biogenesis
Regulation of cell proliferation
Maintenance of cell number
Stem cell population maintenance
Dorsal closure
Border follicle cell migration
Fold Enrichment
B
EC GO Terms
Transmembrane transport
Proteolysis
Oxidation-reduction process
Single-organism metabolic process
Transport
Metabolic process
Fold Enrichment
C
RNAseq (log2)
ISC+EB Expression (log2 POLII Occupancy)
D
RNAseq (log2)
EC Expression (log2 POLII Occupancy)
E
RNAseq (log2)
Dl
mira
esg
N
mew
lin-28
Pvf2
E(spl)mbeta-HLH
polo
sc
Pvr
Atac2
ISC+EB Expression (log2 POLII Occupancy)
F
RNAseq (log2)
apt
CG11247
Sox21a
sc
cyc
Sox100B
z
jumu
ISC+EB Expression (log2 POLII Occupancy)
G
Expressed (%)
High Confidence
Medium Confidence
Low Confidence
Genome-wide RNAi Screen Hits (Zeng et al., 2015)

Figure S1 (related to Fig 1) Comparison to Previous Genome-wide Datasets

A. Significantly enriched gene ontology terms in ISC/EB specific genes.

B. Significantly enriched gene ontology terms in EC specific genes.

C and D. Correlation between POLII DamIDseq and RNAseq data for ISC/EB and EC respectively. E and F. Correlation between POLII DamIDseq and RNAseq in ISC/EBs highlighting known ISC/EB specific genes (E) and transcription factors (F).

G. Comparison DamIDseq expression in ISC/EB to hits from a genome-wide screen of ISC/EB regulators.

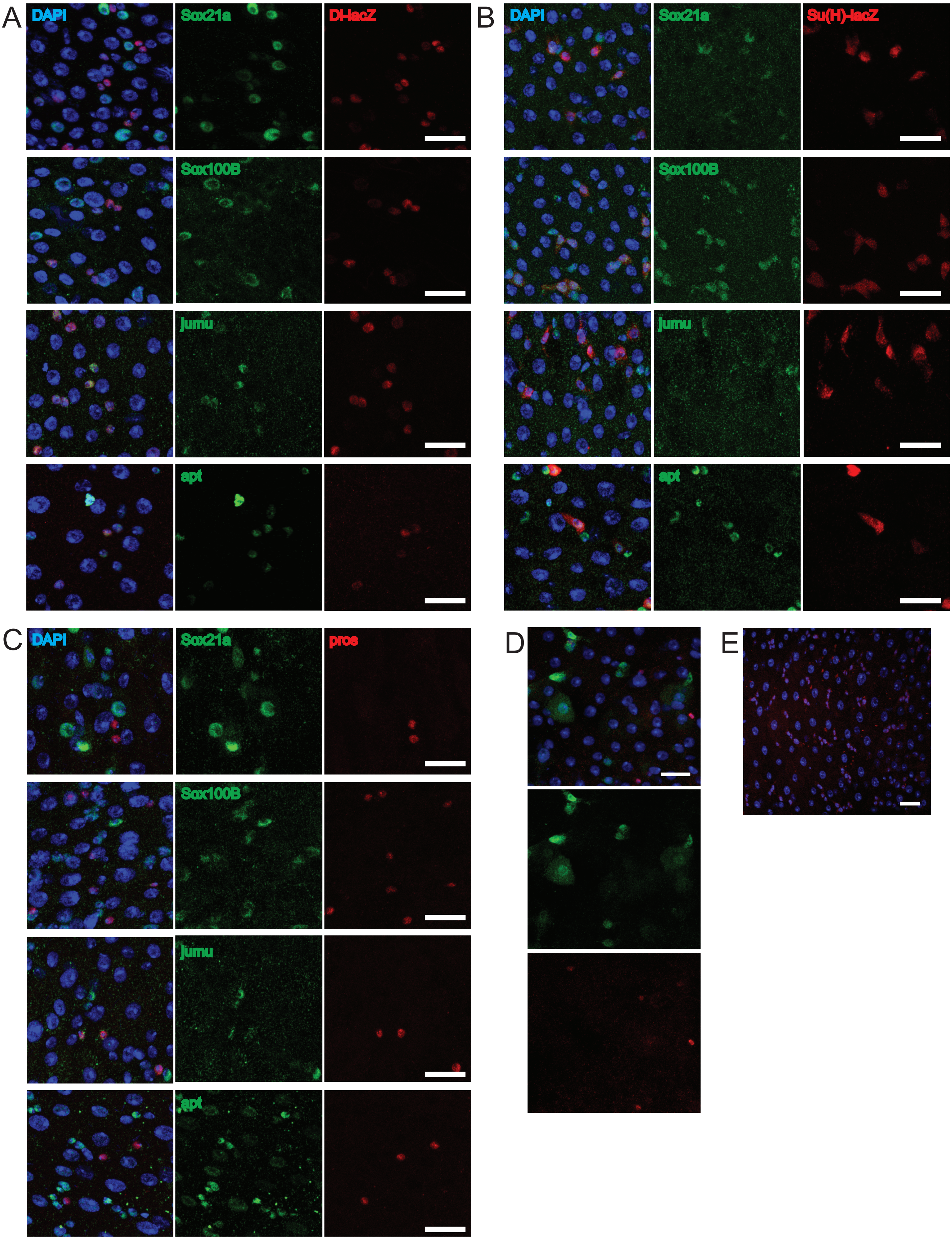

A
DAPI
Sox21a
Dl-lacZ
Sox100B
jumu
apt
B
DAPI
Sox21a
Su(H)-lacZ
Sox100B
jumu
apt
C
DAPI
Sox21a
pros
Sox100B
jumu
apt
D
E

Figure S2 (related to Fig 2) – Expression of Stem/Progenitor Transcription Factors

A - C. Maximum projection Z stacks showing expression of conserved TFs in the midgut. Background signal was subtracted using the remove outliers function (Fiji) and brightness/contrast increased for clarity. DAPI blue, TF green as indicated, A. Dl-lacZ red, B. Su(H)-lacZ red, C. prospero red. Scale bars are 20µm. Quantification is shown in Figure 2F-H.

D. Zfh2-GAL4>UASEGFP shows expression (green) mostly in small ISC/EB cells that are negative for the EE marker prospero (red) and occasional EEs and ECs. Blue is DAPI. Scale bar is 20µm.

E. Zfh2 antibody (Rat 1/500, from Chris Doe) staining shows expression (red) in small cells and not large ECs. Scale bar is 20µm.

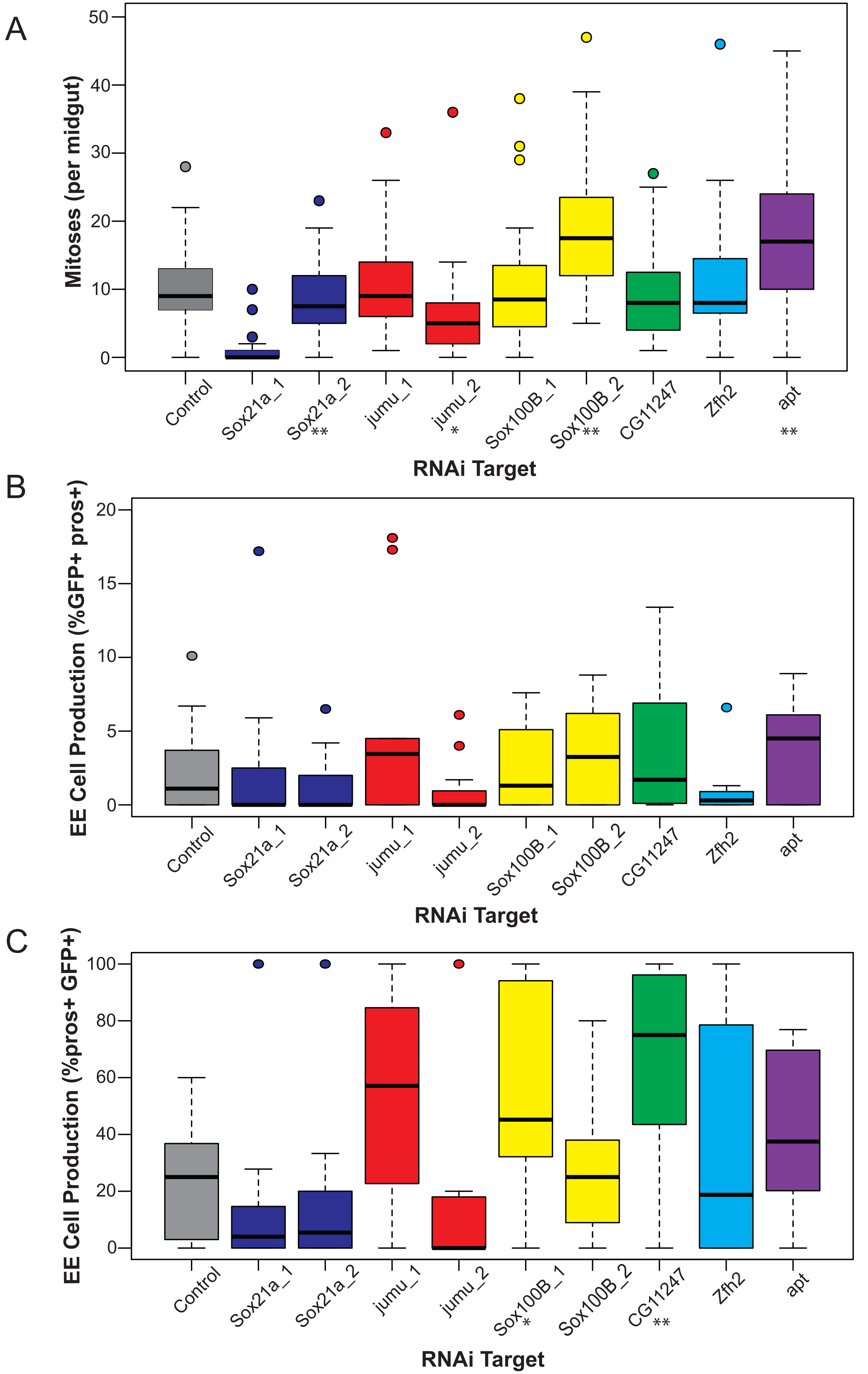
A
Mitoses (per midgut)
50
40
30
20
10
0
Control
Sox21a_1
Sox21a_2
**
jumu_1
jumu_2
*
Sox100B_1
Sox100B_2
**
CG11247
Zfh2
apt
**
RNAi Target
B
EE Cell Production (%GFP+ pros+)
20
15
10
5
0
Control
Sox21a_1
Sox21a_2
jumu_1
jumu_2
Sox100B_1
Sox100B_2
CG11247
Zfh2
apt
RNAi Target
C
EE Cell Production (%pros+ GFP+)
100
80
60
40
20
0
Control
Sox21a_1
Sox21a_2
jumu_1
jumu_2
Sox100B_1
*
Sox100B_2
CG11247
**
Zfh2
apt
RNAi Target

Figure S3 (related to Fig 3) - Function of Stem/Progenitor Derived Transcription Factors

A. Quantification of mitoses per midgut on transcription factor RNAi in ISC/EBs. ($n \geq 23$ midguts) * indicates $p<0.05$, ** indicates $p<0.01$ in 2 tailed Students' T Test.

B. Percentage of GFP positive cells that are prospero positive ($n \geq 9$ midguts)

C. Percentage of total prospero positive cells that are GFP positive. ($n \geq 7$ midguts) * indicates $p<0.05$, ** indicates $p<0.01$ in 2 tailed Students' T Test.

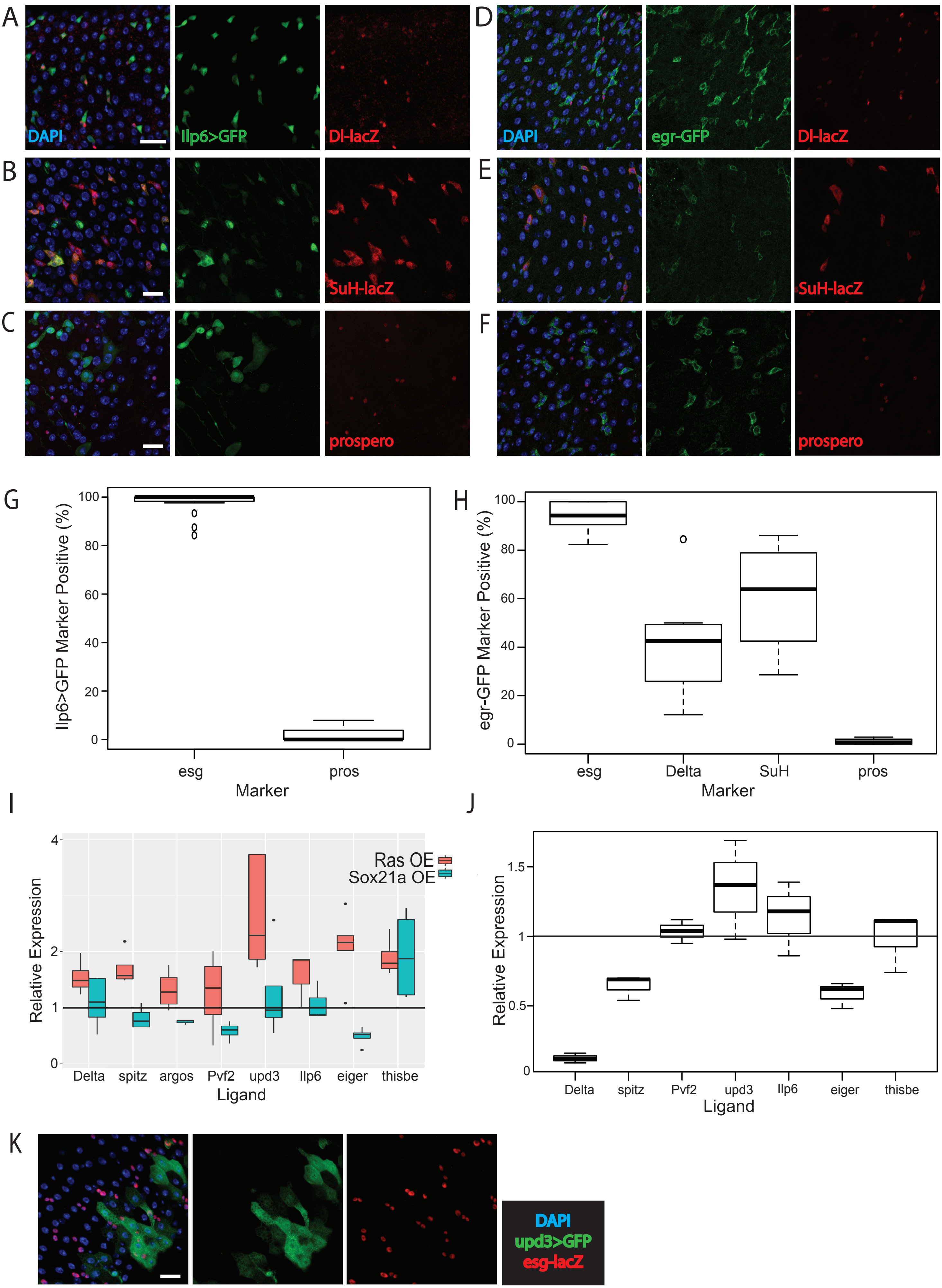

A
DAPI
Ilp6>GFP
Dl-lacZ
B
SuH-lacZ
C
prospero
D
DAPI
egr-GFP
Dl-lacZ
E
SuH-lacZ
F
prospero
G
Ilp6>GFP Marker Positive (%)
100
80
60
40
20
0
esg
pros
Marker
H
egr-GFP Marker Positive (%)
100
80
60
40
20
0
esg
Delta
SuH
pros
Marker
I
Relative Expression
4
2
1
0
Ras OE
Sox21a OE
Delta
spitz
argos
Pvf2
upd3
Ilp6
eiger
thisbe
Ligand
J
Relative Expression
1.5
1
0.5
0
Delta
spitz
Pvf2
upd3
Ilp6
eiger
thisbe
Ligand
K
DAPI
upd3>GFP
esg-lacZ

Figure S4 (related to Fig 4) - ISC/EB ligand expression

A-C. Expression of Ilp6-GAL4 driven EGFP (green) in A. Dl-lacZ marked stem cells, B. Su(H)-lacZ marked EBs and C. prospero positive enteroendocrine cells. Scale bars are 20µm.

D-F. Expression of egr-GFP (green) in D. Dl-lacZ marked stem cells, E. Su(H)-lacZ marked EBs and F. prospero positive enteroendocrine cells. Scale bars are 20µm.

G. Quantification of Ilp6 expression overlap with cell-type markers (n=17 for esg, 10 for prospero).

H. Quantification of egr expression overlap with cell-type markers (n=17, 7, 10 and 8 for esg, Dl, Su(H) and prospero respectively).

I. Whole midgut qPCR of ligand expression in response to Ras overexpression (red) or Sox21a overexpression (blue).

J. Ligand expression by whole gut qPCR following 7 days of Sox21a RNAi.

K. upd3-GAL4 drives GFP (green) expression in both ECs (large nuclei, DAPI, blue) and ISC/EBs (esg-lacZ, red). Scale bar is 20µm.

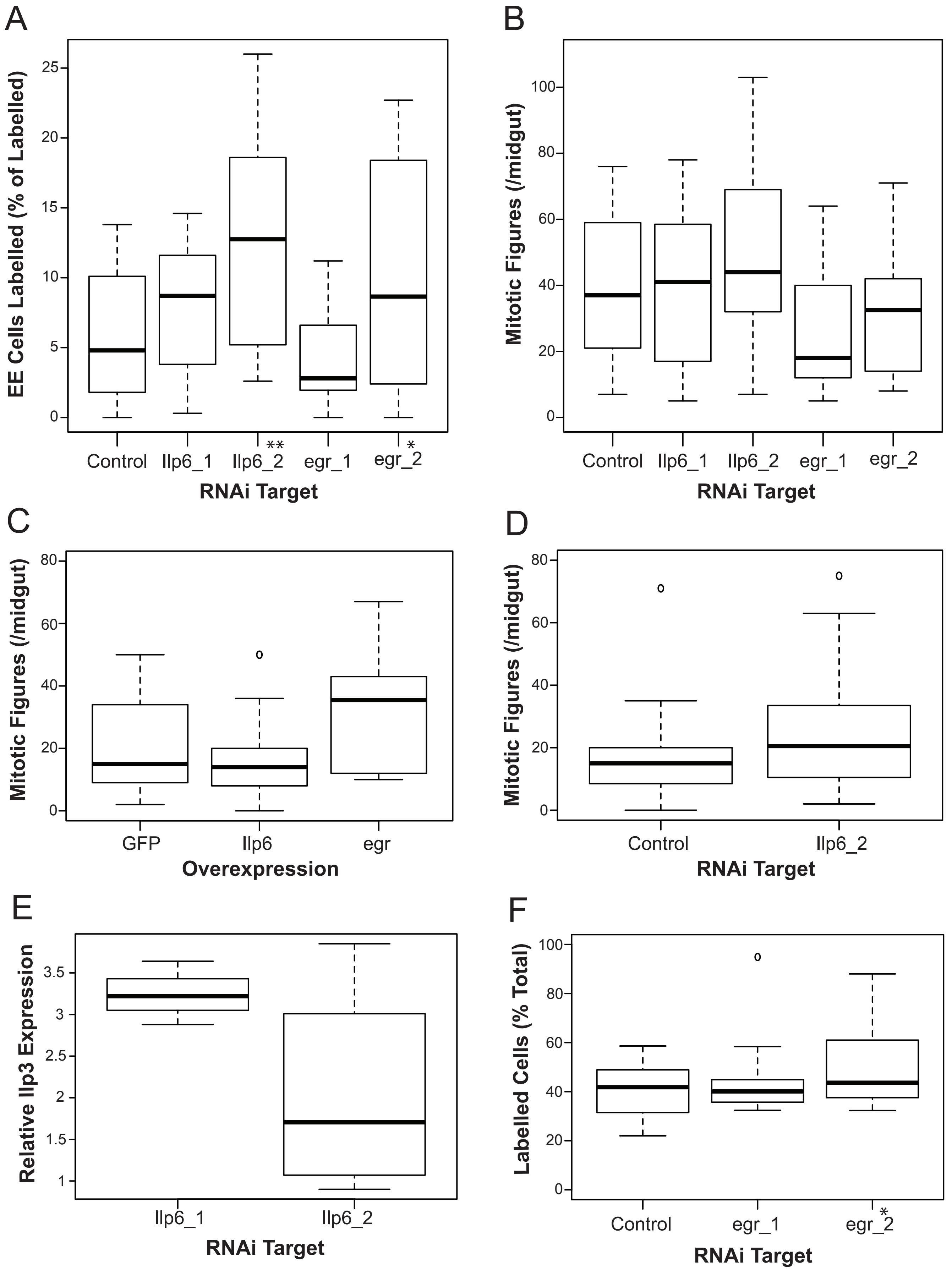
A
EE Cells Labelled (% of Labelled)
Control
Ilp6_1
Ilp6_2
**
egr_1
egr_2
*
RNAi Target
B
Mitotic Figures (/midgut)
Control
Ilp6_1
Ilp6_2
egr_1
egr_2
RNAi Target
C
Mitotic Figures (/midgut)
GFP
Ilp6
egr
Overexpression
D
Mitotic Figures (/midgut)
Control
Ilp6_2
RNAi Target
E
Relative Ilp3 Expression
Ilp6_1
Ilp6_2
RNAi Target
F
Labelled Cells (% Total)
Control
egr_1
egr_2
*
RNAi Target

Figure S5 (related to Fig 5) - Ilp6 and egr regulate homeostasis

A. EE cell production on Ilp6 and egr knockdown ($n \geq 15$).

B. Mitoses per midgut on Ilp6 and egr knockdown in ISC/EBs ($n \geq 16$).

C. Mitoses per midgut on Ilp6 and egr overexpression in ISC/EBs ($n \geq 10$).

D. Mitoses per midgut on organism-wide knockdown of Ilp6 ($n \geq 24$).

E. Whole midgut qPCR shows upregulation of Ilp3 when Ilp6 is knocked down.

F. Lineage tracing of egr RNAi ($n \geq 15$) (Control repeated from Figure 5C, as Ilp6 and egr kd experiments were performed together).
